# Prevalence and determinants of chewing khat among women in Ethiopia: data from Ethiopian demographic and health survey 2016

**DOI:** 10.1186/s12888-021-03136-y

**Published:** 2021-03-04

**Authors:** Yimenu Yitayih, Jim van Os

**Affiliations:** 1 Department of Psychiatry, College of Medical faculty, Jimma University, Jimma, Ethiopia; 2 Department of Psychiatry, UMC Utrecht Brain Center, University Medical Center Utrecht, Utrecht University, Utrecht, The Netherlands; 3 Department of Psychiatry and Neuropsychology, School for Mental Health and Neuroscience, Maastricht University Medical Centre, Maastricht, the Netherlands; 4 Department of Psychosis Studies, Institute of Psychiatry, Psychology & Neuroscience, King’s College London, London, UK

**Keywords:** Ethiopia, Substance-related disorders, *Catha edulis*, Khat use, Women’s health, Community health

## Abstract

**Background:**

In Ethiopia and other countries in eastern Africa, khat abuse is an increasing public health problem. Levels of use appear to be increasing in women, who are more vulnerable to khat-related problems. However, population-based data are lacking as studies have been small and related to specific settings. This study aimed to contribute to current knowledge on the prevalence of chewing khat and associated factors among women in Ethiopia, using data from the 2016 Ethiopian national demographic and health survey.

**Methods:**

The 2016 EDHS used a two-stage stratified sampling design to select households. A total of 645 enumeration areas (202 urban and 443 rural) were selected, based on the 2007 Ethiopia Population and Housing Census. In these, 18,008 households were considered, from which 15,683 women were included from individual households. The women were interviewed by trained lay interviewers. Data were tabulated and logistic regression was used to examine mutually adjusted associations, expressed as adjusted odds ratios.

**Results:**

The lifetime prevalence of chewing khat among women was 9.9%. Current khat use was 8.4%, with a mean of 14.2 days of use in the last month. Khat use increased with increasing age, remaining constant after age 35 years, having one child, lower educational level, being Muslim by religion and not pertaining to the lowest wealth index category. Not being in a marital relationship with the most recent sex partner and Protestant religion were protective factors.

**Conclusion:**

Lifetime prevalence of chewing khat among women in Ethiopia is substantial and associated with specific sociodemographic risks. These can be used in targeted public health efforts to control the use of khat and reduce the associated health and economic burden.

## Background

Khat (*Catha edulis*) is a natural stimulant widely cultivated and available in East Africa and the Middle East [1]. The biochemically active ingredients of khat are cathinone and cathine, which are alkaloid chemicals bearing resemblance to the psychoactive substance of amphetamine, both structurally and functionally [2]. Cathinone is the main psychoactive component of khat leaves and a potent stimulator of the sympathetic nervous system as well as the central nervous system, similar to the pharmacological effects of amphetamine [2]. Chewing fresh leaves of the khat tree is the most common mode of intake [3].

Khat abuse is an increasing public health problem and strongly associated with adverse mental and other outcomes and such as psychological distress, poorer quality of life and increased road traffic accident in low-income countries [4]. Across the world, an estimated 20 million people chew khat leaves daily [4]. Khat is generally used for its perceived ability to facilitate interpersonal communication in social settings, to induce euphoric and performance enhancing effects and to fend off fatigue, as well as for its perceived medicinal value for the treatment of headaches and common cold [5].

Khat use has been associated with numerous health problems. Basic research studies have found evidence of altered stress response [6], cognitive deficits [7, 8], increased levels of depressive symptoms and distress [9] and insomnia [10] in habitual khat users. Research has also shown that prolonged exposure to khat may lead to a long-lasting sensitization to the effects of other drugs [11]. In addition to physical and mental harm, much time and household income is spent on obtaining and chewing khat [12], which severely affects users’ social life and family [13]. The habit of chewing khat may be postulated as one of the risky behaviours that could fuel the spread of sexually transmitted infections (STIs) due to associated risky sexual behaviours like having casual sex, unprotected sex and early initiation of sexual activity reported among chewers [14]. Studies reveal that khat consumption may impact fetal health, for example affecting fetal growth by inhibiting utero-placental blood flow [15, 16].

Research shows that Khat use is a common practice in Ethiopia [17]. Before 2000, khat chewing by women was generally not considered acceptable [18]. Currently, however, use of khat among women is increasing very rapidly [19]. Research suggests that husbands may be in part responsible for these changes as they encourage their wives to share in the chewing of khat [18].

People who use khat are more vulnerable to khat-related problems like depressive symptoms, posttraumatic stress disorder and common mental disorder [20, 21], the issue to date has not received much attention from local authorities.

Few studies have been conducted to establish prevalence of use and associated factors, and these tended to be small and focusing on specific settings [22].

The current investigation, therefore, aimed to contribute to current knowledge on the use of khat and associated factors among women in Ethiopia, using data from the uniquely representative and population-based 2016 Ethiopian national demo-graphic and health survey (EDHS).

## Methods

### Study design and sampling

The 2016 EDHS used a two-stage stratified sampling design to select households. In the first stage, there were 645 enumeration areas (202 urban and 443 rural) based on the 2007 Ethiopia Population and Housing Census (PHC). A total of 18,008 households were considered, of which 16,650 households and 15,683 women were eligible. The women were interviewed by trained lay interviewers. All women at reproductive age (15 to 49 years) who were either permanent residents of the selected households or visitors who stayed in the selected household the night before the survey, were eligible for the study. A total of 15,683 women aged 15–49 years were interviewed with a response rate of 95%. The EDHS was designed to provide sociodemographic and health indicators at national (urban and rural) and regional levels. The survey follows an international methodological approach and is conducted at five-year intervals. Detailed methodology is found elsewhere [23].

### Measurements

The dependent variable was chewing khat in the last 30 days. Women who chewed khat in the last month were considered as current khat user. Sociodemographic, socioeconomic and sex-related variables associated with risk were used as independent variables. The independent variables included in this analysis were age, educational status, and marital status.

Household wealth index is based on household ownership of consumer items such as a fan, television, and car. In addition, dwelling characteristics, such as the flooring material; type of drinking water source; and toilet facilities, are among other characteristics related to wealth.

It has five categories: poorest, poorer, middle, richer, and richest), residence, religion, residence, sex of household head (person in the house with main responsibility for making decisions and providing income; can be female regardless of presence of males in the household), number of children, number of sexual partners (if participants were not willing to disclose the information, the variable was coded ‘9’ as a category for ‘not known’), sexual transmitted infection (STI) in the last 12 months, relationship to household head, ever reads newspaper or magazine (yes/no), ever listens to radio (yes/no), ever watches TV (yes/no), ever use of internet (yes/no), most recent sex partner (if participants indicated they had no sexual partner, this was coded as a separate category; if the participant was not willing to disclose the information, the variable was coded ‘9’ as a separate category for ‘not known’).

### Statistical data analysis

The data were extracted, edited, and analysed using SPSS version 23 for Windows. Frequency tables were used to summarize sociodemographic characteristics and prevalence of khat use. Bivariate logistic regression was performed separately for each independent variable. Independent variables with a *p*-value< 0.05 were entered into the final model for multivariable analysis. Variables in the mutually adjusted multivariable model with a two-sided *p*-value< 0.05 were considered statistically significant. In order to check for multicollinearity among variables in the multivariable model, variance inflation factors were calculated for all independent variables.

## Results

A total of 15,683 participants were included. The mean (SD) age of women was 27.9+ (9.2) years. The majority were Orthodox Christian by religion (40.9%), had no education (44.8%) and were from rural settings (65.9%) (Table 1). The highest proportion were from the age group 15–19 years (22.3%) followed by the 20–24 years group (18.5%) and the 25–29 years group (18.1%). A total of 24.8% of participants were identified as ‘poorest’ and 13% as ‘poorer’ according to the wealth index. The mean number of children ever born was 2.6.

**Table 1:** Characteristic of the study participants of khat chewing among women in Ethiopia

Variables		Total		Current khat chewing			
		n	%	No		Yes	
				N	%	n	%
Age group in years	15–19	3498	22.3	3375	96.5	123	3.5
	20–24	2903	18.5	2717	93.6	186	6.4
	25–29	2845	18.1	2586	90.9	259	9.1
	30–34	2241	14.3	1964	87.6	277	12.4
	35–39	1917	12.2	1708	89.1	209	10.9
	40–44	1302	8.3	1134	87.1	168	12.9
	45–49	977	6.2	878	89.9	99	10.1
Religion	Orthodox	6413	40.9	6231	97.2	182	2.8
	Catholic	91	0.6	89	97.8	2	2.2
	Protestant	2814	17.9	2810	99.9	4	0.1
	Muslim	6209	39.6	5078	81.8	1131	18.2
	Traditional	84	0.5	82	97.6	2	2.4
	Other	72	0.5	72	100	0	0
Educational level	No education	7033	44.8	6310	89.7	723	10.3
	Primary	5213	33.2	4780	91.7	433	8.3
	Secondary	2238	14.3	2128	95.1	110	4.9
	Higher	1199	7.7	1144	95.4	55	4.6
Residence	Urban	5348	34.1	4876	91.2	472	8.8
	Rural	10,335	65.9	9486	91.8	849	8.2
Household head	Male	10,853	69.2	9901	91.2	952	8.8
	Female	4830	30.8	4461	92.4	369	7.6
Relationship to household head	Head	2803	17.9	2536	90.5	267	9.5
	Wife	7413	47.3	6618	89.3	795	10.7
	Daughter	3761	24.0	3582	95.2	179	4.8
	Grand daughter	178	1.1	174	97.8	4	2.2
	Mother	28	0.2	25	89.3	3	10.7
	Sister	366	2.3	345	94.3	21	5.7
	Other relative	621	3.9	595	95.8	26	4.2
	Not relative	513	3.3	487	94.9	26	5.1
Reads newspaper/magazine	No	13,106	83.6	11,936	91.1	1170	8.9
	Yes	2577	16.4	2426	94.1	151	5.9
Listens to radio	No	10,338	65.9	9433	91.3	905	8.7
	Yes	5345	34.1	4929	92.2	416	7.8
Watches TV	No	10,084	64.3	9226	91.5	858	8.5
	Yes	5599	35.7	5136	91.7	463	8.3
Uses internet	No	14,329	91.4	13,089	91.4	1240	8.6
	Yes	1354	8.6	1273	94.0	81	6.0
Wealth index	Poorest	3874	24.8	3664	94.1	230	5.9
	Poorer	2046	13.0	1804	88.2	242	11.8
	Middle	2002	12.8	1807	90.3	195	9.7
	Richer	2042	13.0	1893	92.7	149	7.3
	Richest	5699	36.3	5194	91.1	505	8.9
Any STI last 12 months	No	15,600	99.5	14,286	91.6	1314	8.4
	Yes	83	0.5	76	91.6	7	8.4
Children	None	5409	34.5	5167	95.5	242	4.5
	1	1974	12.6	1811	91.7	163	8.3
	2	1704	10.9	1549	90.9	155	9.1
	≥3	6596	42.0	5835	88.5	761	11.5
Number of sexual partner	None	1282	8.2	1213	94.6	69	5.4
	1	9178	58.5	8387	91.4	791	8.6
	2	2207	14.1	2021	91.6	186	8.4
	≥3	577	3.7	530	91.8	47	8.2
	Not known	2439	15.5	2211	90.7	228	9.3
Most recent sex partner	Spouse	9203	58.7	8396	91.2	807	8.8
	Other	691	4.4	657	95.1	34	4.9
	No partner	162	1.0	151	93.2	11	6.8
	Not known	5627	35.9	5158	91.7	469	8.3
Life time khat use	No	14,130	90.1	–	–	–	–
	Yes	1553	9.9	–	–	–	–
Current khat chewing	No	14,366	91.6	–	–	–	–
	Yes	1317	8.4	–	–	–	–

*STI Sexual transmitted infection;

The lifetime prevalence of khat chewing among women was 9.9%. The prevalence of current khat chewing was 8.4%, with a mean number of 14.2 days of use in the last month. Highest prevalence of current khat chewing was seen in the age group 40–44 years old (12.9%), 30–34 years old (12.4%), 35–39 years old (10.9%) and 45–49 years old (10.1%).

Prevalence of current khat chewing was lowest among participants from the lowest wealth index (5.9%), compared to participants from other wealth index categories. Higher prevalence of current khat chewing was also seen among individuals without education (10.3%) or only primary education (8.3%), compared to those with secondary education (4.9%) and higher education (4.6%). Among current khat chewers, the prevalence was highest among those of Muslim religion (18.2%) as compared to other religious groups (0.0–2.8%). The highest prevalence of khat chewing was seen when the relationship to the household head was wife (10.7%), and mother (10.7%), followed by head (9.5%). Prevalence of current khat use was also higher among respondents who had a single sexual partner (8.6%), two partners (8.4%) and three or more partners (8.2%), compared to those with no sexual partner (5.4%). Prevalence of khat use was similar regardless of STI in the last 12 months. The prevalence of current khat use was 8.8% in urban areas versus 8.2% in rural areas, and rates were slightly higher among those not reading newspaper/magazines (8.9% versus 5.9% of those reading these), those not listening to radio (8.7% versus 7.8% of those listening to radio), those not watching TV (8.5% versus 8.3% of those watching), and those without access to internet (8.6% versus 6.0% of those with access).

### Multivariable analysis

Table 2 presents univariable and multivariable models of current khat use. Most predictors contributed in the univariable models. The multivariable model revealed that khat use increased with increasing age until age 35 years, after which it remained more or less constant, having one child (adjusted odds ratio [AOR]: 1.63; 95% CI, 1.19–2.24), lower educational level (AOR: 2.32; 95% CI, 1.40–3.87), being Muslim by religion (AOR: 10.48; 95% CI, 8.41–13.06) and not pertaining to the lowest wealth index category. Protective factors were not being in a marital relationship with the sex partner (AOR: 0.55; 95% CI, 0.37–0.80) and Protestant religion (AOR: 0.06; 95% CI, 0.01–0.19). There was no evidence of multicollinearity among variables in the multivariable model as the mean variance inflation factor was 1.46 and the highest was 2.66, i.e. much lower that the ‘problematic’ range of 5–10.

**Table 2:** Logistic regression analysis of factors associated with khat chewing among women in Ethiopia, Ethiopia Demographic and Health Survey (EDHS), 2016 (*N* = 15,683)

Variable		cOR	95% CI	*P-*value	aOR	95% CI	*P-*value
Age group in years	15–19	Ref					
	20–24	1.87	1.48–2.37	< 0.001	1.84	1.30–2.60	< 0.001*
	25–29	2.74	2.20–3.42	< 0.001	2.53	1.75–3.64	< 0.001*
	30–34	3.87	3.10–4.82	< 0.001	4.28	2.91–6.30	< 0.001*
	35–39	3.35	2.66–4.22	< 0.001	3.47	2.30–5.24	< 0.001*
	40–44	4.06	3.19–5.17	< 0.001	5.27	3.45–8.06	< 0.001*
	45–49	3.09	2.35–4.07	< 0.001	3.48	2.19–5.53	< 0.001*
Religion	Orthodox	Ref					
	Protestant^a^	0.07	0.03–0.15	< 0.001	0.06	0.01–0.19	< 0.001*
	Muslim	7.63	6.49–8.95	< 0.001	10.48	8.41–13.06	< 0.001*
	Traditional	0.83	0.20–3.42	0.80	2.41	0.56–10.30	0.234
Educational level	No education	2.38	1.79–3.15	< 0.001	1.63	0.96–2.77	0.068
	Primary	1.88	1.41–2.51	< 0.001	2.32	1.40–3.87	0.001*
	Secondary	1.07	0.77–1.49	1.47	1.49	0.89–2.50	0.125
	Higher	Ref					
Wealth index	Poorest	Ref					
	Poorer	2.13	1.76–2.57	< 0.001	2.87	2.23–3.68	< 0.001*
	Middle	1.71	1.40–2.09	< 0.001	2.38	1.82–3.11	< 0.001*
	Richer	1.25	1.01–1.55	0.04	1.92	1.43–2.57	< 0.001*
	Richest	1.54	1.32–1.82	< 0.001	2.85	2.23–3.65	< 0.001*
Residence	Urban	Ref					
	Rural	0.92	0.82–1.04	0.192			
Household head	Male	Ref					
	Female	0.86	0.75–0.97	0.02	0.96	0.68–1.36	0.832
Children	None	Ref					
	1	1.92	1.56–2.36	< 0.001	1.63	1.19–2.24	0.002*
	2	2.13	1.73–2.63	< 0.001	1.34	0.94–1.90	0.098
	≥3	2.78	2.39–3.23	< 0.001	1.14	0.81–1.61	0.430
Number of sexual partners	None	Ref					
	1	1.65	1.28–2.13	< 0.001	0.74	0.55–1.01	0.060
	2	1.62	1.21–2.15	0.001	1.02	0.83–1.25	0.842
	≥3	1.55	1.06–2.28	0.024	1.11	0.75–1.63	0.591
	Not known	1.81	1.37–2.39	< 0.001	0.63	0.45–0.90	0.010
STI last 12 months	No	Ref					
	Yes	1.00	0.46–2.17	1.00	0.82	0. 35–1.96	0.670
Relationship to household head	Head	Ref					
	Wife	1.14	0.98–1.32	0.077	1.27	0.85–1.90	0.239
	Daughter	0.47	0.39–0.57	< 0.001	0.97	0.66–1.41	0.885
	Grand daughter	0.21	0.08–0.59	0.003	0.51	0.11–2.25	0.381
	Mother	1.13	0.34–3.80	0.831	2.72	0.69–10.75	0.152
	Sister	0.57	0.36–0.91	0.019	0.87	0.44–1.73	0.707
	Other relative	0.41	0.27–0.62	< 0.001	0.91	0.50–1.62	0.752
	Not relative	0.50	0.33–0.76	0.001	0.92	0.49–1.73	0.806
Reads newspaper or magazine	No	Ref					
	Yes	0.63	0.53–0.75	< 0.001	0.91	0.68–1.23	0.570
Listens to radio	No	Ref					
	Yes	0.87	0.77–0.99	0.038	1.16	0.96–1.40	0.103
Watches TV	No	Ref					
	Yes	0.96	0.86–1.09	0.605			
Use of internet	No	Ref					
	Yes	0.67	0.53–0.84	0.001	1.21	0.81–1.81	0.332
Most recent sex partner	Spouse	Ref					
	Other	0.53	0.37–0.76	0.001	0.55	0.37–0.80	0.002 *
	No partner	0.76	0.41–1.40	0.378	0.79	0.37–1.70	0.551
	Not known	0.94	0.84–1.06	0.360	0.97	0.81–1.17	0.781

*cOR* Crude odds ratio, *aOR* Adjusted odds ratio, * *P* < 0.05, STI Sexually transmitted infection

^a^includes small group of Catholics

## Discussion

To the best of our knowledge, this was the first reported study that investigated the prevalence of khat chewing and associated factors among women in the Ethiopian population using the latest nationally representative data obtained from EDHS 2016. The findings reveal that in women, the life time prevalence of khat chewing is 9.9% versus 8.4% of current use. Multivariable analysis revealed that current use was more prevalent with higher age, having one child, lower educational level, being Muslim by religion and not pertaining to the lowest wealth index category. Protective factors were not being in marital relationship with sex partner and Protestant religion.

The prevalence of current khat use among women in this study, at 8.4%, was lower as the rate reported in Yemen (29.6%) [18]. Cultural factors underlying use may differ between the two countries, but differences may also be due to variation in survey design, and other methodological issues.

The multivariable model revealed that khat use increased with age until age 35 years, after which it remained approximately constant. This is consistent with a study conducted in eastern Ethiopia [24]. The possible reason may be that as age increases, women are more likely to have alterations in life circumstances such as bereavement, social isolation, lack of social support and financial difficulties, all of which have been found to increase the risk of substance use [25, 26]. It has also been observed that depressive symptoms may increase during the transitional phase from peri-menopause to post-menopause [27, 28], possibly driving self-medication strategies. Another reason may be that younger women tend to be more under family control, which likely reduces risk of exposure.

Not pertaining to the lowest wealth index category was associated with higher khat use rates. One obvious reason is that buying khat requires a certain level of income [29]. Furthermore, chewing khat is often developed as part of doing business in higher income groups [13]. Also, khat chewing practices affect the economic status of society due to the fact that collectively, it contributes to the loss of thousands of acres of arable land and billions of hours of work as well as utilization of scarce resources to buy khat rather than acquiring nutritious foods and care for household members [30].

Those pertaining to the religion of Islam were at higher odds to chew khat as compared to those of Orthodox religion. This is consistent with earlier work in Ethiopia [31, 32]. Chewing khat is a common practice and traditionally accepted in Islamic communities, associated with perceived benefits in concentration during work and prayer time [33, 34].

Women with lower educational status had more risk of current khat chewing compared to women with higher educational status. The current study was in agreement with a study conducted in the Jazan region, in Saudi Arabia, which showed that illiteracy was associated with higher odds of chewing khat [35]. A possible reason is that a lower level of education is associated with lack of knowledge about the negative consequences of khat use [35].

Furthermore, women who had one child had higher odds of khat chewing as compared to women without children. A possible reason is joint use under the influence of a stable partner using khat. However, this would then only apply to the period of the first child, as women with more children again had reduced risk for chewing khat. One explanation is that with an increasing number of children, less resources would be available for the khat habit.

Given that khat use among women is prevalent, and with a mean number of 14.2 days of use in the last month, information campaigns are required in order to inform the public about harmful effect of khat use [36]. For those who are affected by khat use-related disorder and comorbid conditions, treatment services should be provided. Addiction services can be offered using the existing health system. For example, primary health care may introduce routine screening and treatment for substance use [36, 37].

However, given that the majority of people do not have access to good quality of care; it is a timely need for the sustainable programs [38]. Therefore, it is a timely need for pertinent stakeholders of the Ethiopian public health care system to introduce novel approaches to generate financially sustainable programs for the early diagnosis, treatment and control of addiction through a group of well-trained health care providers.

### Limitations

The results should be interpreted in the light of several limitation. First, this was a secondary data analysis which overlooked key variables that are relevant for khat use such as availability, affordability, family substance use and awareness of health consequences of khat use. Second, we cannot exclude social desirability-related underreporting of khat use behaviour, given that female khat use is still associated with a degree of stigma in Ethiopian society.

## Conclusion

In conclusion, the lifetime rate of chewing khat among women in Ethiopia is substantial and associated with specific sociodemographic risks. These can be used in targeted public health efforts to control increasing levels of khat use among women and reduce the associated health and economic burden.

## Data Availability

The datasets generated and analyzed during the current study will be available in the public repository.

## Abbreviations

AOR: Adjusted odds ratio
CDC: Centers for Disease Control and Prevention
EDHS: Ethiopian national demo-graphic and health survey
STIS: Sexually transmitted infections
PHC: Population and Housing Census
